# Artificial intelligence-assisted interpretation of Ki-67 expression and repeatability in breast cancer

**DOI:** 10.1186/s13000-022-01196-6

**Published:** 2022-01-30

**Authors:** Lina Li, Dandan Han, Yongqiang Yu, Jinze Li, Yueping Liu

**Affiliations:** Department of Pathology, The Fourth Hospital of Hebei Medical University, No.12 Jiankang Road, Shijiazhuang, Hebei 050011 People’s Republic of China

**Keywords:** Breast cancer, Ki-67, Artificial intelligence, Ki-67 standard reference card, Repeatability

## Abstract

**Background:**

Ki-67 standard reference card (SRC) and artificial intelligence (AI) software were used to evaluate breast cancer Ki-67LI. We established training and validation sets and studied the repeatability inter-observers.

**Methods:**

A total of 300 invasive breast cancer specimens were randomly divided into training and validation sets, with each set including 150 cases. Breast cancer Ki-67 standard reference card ranging from 5 to 90% were created. The training set was interpreted by nine pathologists of different ages through microscopic visual assessment (VA), SRC, microscopic manual counting (MC), and AI. The validation set was interpreted by three randomly selected pathologists using SRC and AI. The intra-group correlation coefficient (ICC) were used for consistency analysis.

**Results:**

In the homogeneous and heterogeneous groups of validation sets, the consistency among the pathologists that used SRC and AI was very good, with an ICC of>0.905. In the validation set, using SRC and AI, three pathologists obtained results that were very consistent with the gold standard, having an ICC above 0.95, and the inter-observer agreement was also very good, with an ICC of>0.9.

**Conclusions:**

AI has satisfactory inter-observer repeatability, and the true value was closer to the gold standard, which is the preferred method for Ki-67LI reproducibility; While AI software has not been popularized, SRC may be interpreted as breast cancer Ki-67LI’s standard candidate method.

## Background

Ki-67 is an indispensable nuclear antigen for cell proliferation. It can be rapidly detected using immunohistochemistry [1]. Pathologists generally use visual assessment or manual cell counting under a microscope to evaluate Ki-67LI. However, visual assessment lacks repeatability among observers [2–4]. In manual counting, at least 500–1000 tumor cells must be counted to achieve an acceptable error rate and corrected for heterogeneity, which is a time-consuming and error-prone process [5, 6]. Many studies have shown significant variability among observers in the evaluation of Ki-67LI for breast cancer [7–11], which leads to limitations in its clinical application.

In recent years, digital pathology has made great progress in image acquisition and digital analysis, which makes artificial intelligence comparable to visual evaluation under the microscope regarding Ki-67 interpretation [12]. By comparing the difference between AI-assisted and manual interpretation, the clinical applicability of AI-assisted interpretation was analyzed to provide a scientific theoretical basis for an accurate and individualized treatment of breast cancer patients.

## Material and methods

### Patient cohort and data preparation

We collected 300 cases of invasive breast cancer who underwent modified radical surgical resection of breast cancer in the Fourth Hospital of Hebei Medical University from October 2017 to October 2019, with complete pathological data. A total of 150 cases were used to establish the training set, and another 150 cases were used to build validation sets. All patients were female and had received no neoadjuvant therapy before surgery.

Microscopic hematoxylin and eosin (H&E) staining sections and Ki-67 immunohistochemical staining sections were independently reviewed by two senior breast pathologists. Based on the consensus reached by the two breast pathologists through visual evaluation, Ki-67 expression was visually classified as homogeneous or heterogeneous according to the distribution of Ki-67 positive cells in the sections. Ki-67 is defined as homogenous when it is uniformly expressed throughout the tumor. In contrast, heterogeneity is defined when regions of high proliferation and low proliferation are identified throughout the tumor.

### Develop Ki-67 standard reference card

**Fig. 1:**
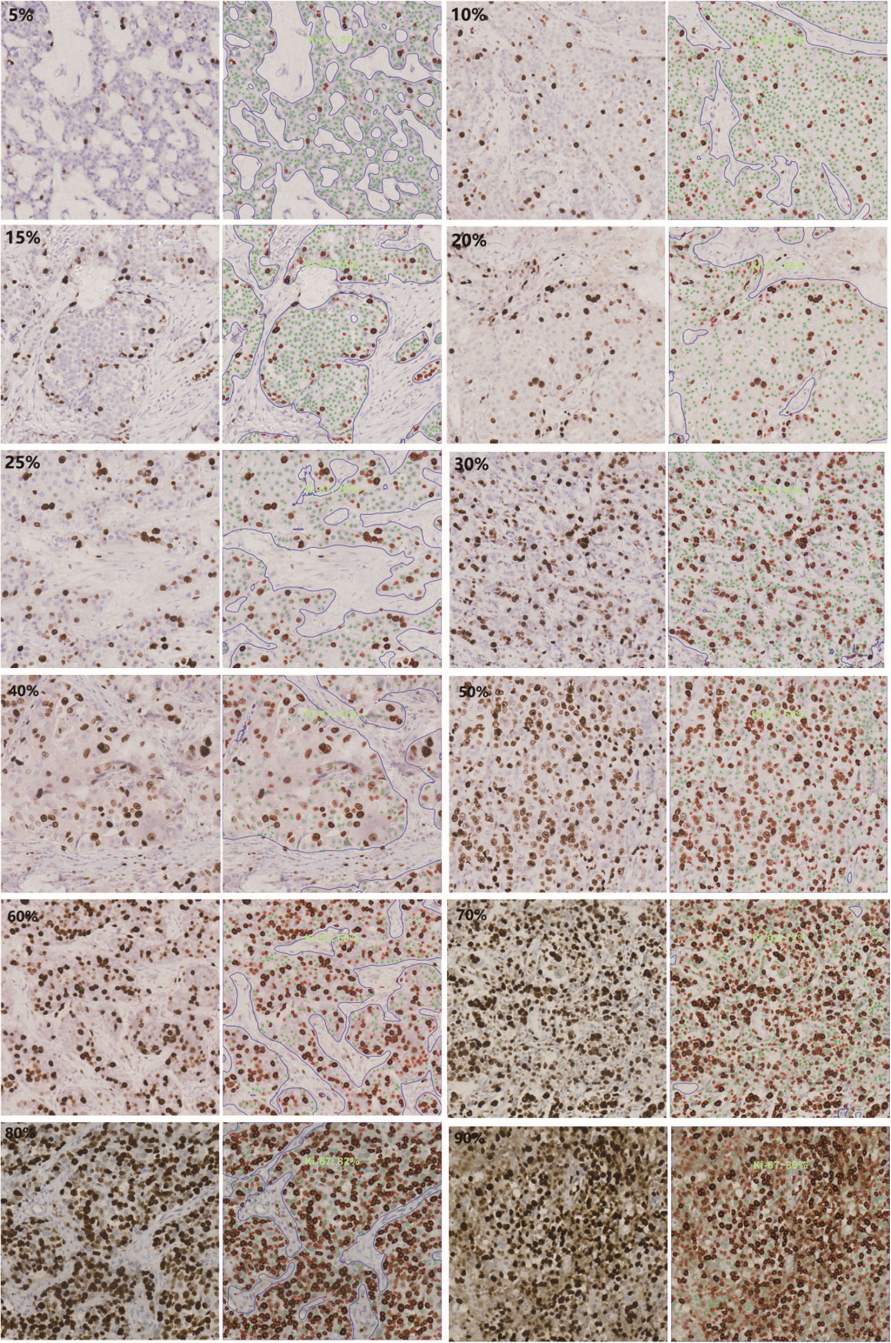
Breast cancer Ki-67 standard comparison card (5, 10, 15, 20, 25, 30, 40, 50, 60, 70, 80, 90%)

Using the PRECICE 600 fully automatic digital slice scanner, 150 cases from the training set were digitally scanned. According to the recommendations of the International Breast Cancer Ki-67 Working Group [5], in the artificial intelligence software, we selected a 550 × 550 μm interpretation frame in the denser tumor cell area, and then AI automatically counted cells in the interpretation frame. The number of positive tumor cells and the total number of tumor cells were reviewed by three breast pathologists who were blinded to the original interpretation. The results of Hida et al. showed that immunohistochemical staining of breast cancer Ki-67 at 10% intervals is a candidate for standard methods [13]. However, the Ki-67 cutoff values for breast cancer ranges from 10 to 30%.Therefore, we set the reference card at intervals of 5% below 30%, at intervals of 10% above 30%, and finally selected Ki-67LI of 5, 10, 15, 20, 25, 30, 40, 50, 60, 70, 80, and 90% as Ki-67 standard reference cards (Fig. 1).

### Ki-67 index assessment

**Fig. 2:**
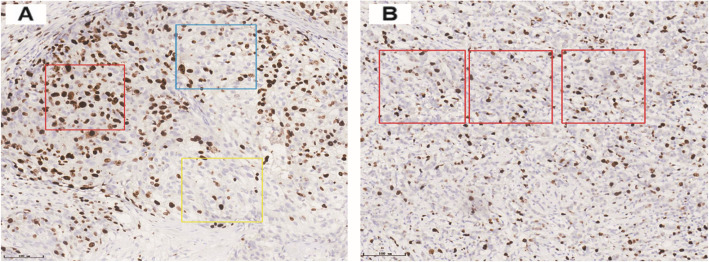
Artificial intelligence software reading frame. A: In heterogeneous tumors,200 × 200 μm reading frame (20×), Red frame covers the hot spot area; Yellow frame covers the cold spot area; Blue frame covers the middle value-added area;B: In homogeneous tumor, 200 × 200 μm reading frame (20×)

In homogeneous tumors, three randomly selected regions were evaluated; and in heterogeneous tumors, hot spots (highest proliferation active regions), cold spots (lowest proliferative active regions), and intermediate proliferative active regions were respectively selected for evaluation. The number of tumor cells covered by the interpretation frame in different areas was different. The 100 × 100, 200 × 200, and 300 × 300 μm interpretation frame covered approximately 60, 200, and 600 tumor cells, respectively. Based on the selection of the area and number of tumor cells covered by different interpretation frames, three 200 × 200 μm interpretation frame were placed in the hot spots, cold spots (lowest proliferation active areas), and intermediate proliferation areas in heterogeneous tumors. In homogeneous tumors, three 200 × 200 μm interpretation frames were randomly placed (Fig. 2 A,B).

### Gold standard

**Fig. 3:**
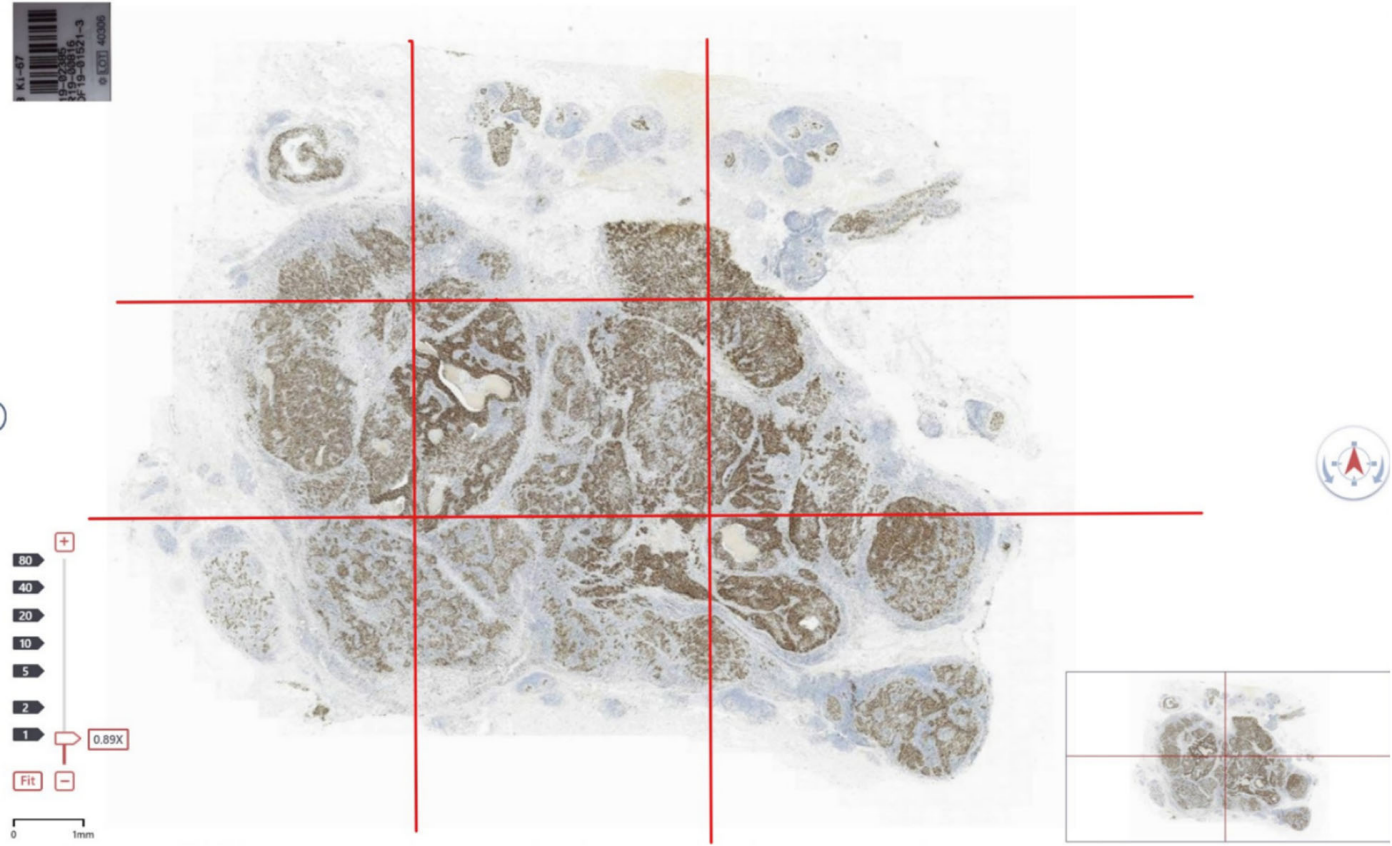
Whole slide imaging (WSI). Each digital slice was divided into 9 areas, and AI automatically counted the number of positive tumor cells and the total number of tumor cells in each area

This research combined artificial intelligence and manual methods to set the gold standard. In 300 digital scanning slices, each digital slice was divided into 9 areas, and AI automatically counted the number of positive tumor cells and the total number of tumor cells in each area (Fig. 3). Three breast cancer pathologists reviewed the number of tumor cells in each area. Finally, the ratio of the number of Ki-67 nuclear positive cells and the total number of tumor cells in each region (M Ki-67) was counted, and M Ki-67 was defined as the research gold standard.

### Interpretation method

#### Training set

9 pathologists of different ages (primary pathologists (diagnosis time < 3 years) A, B, C, intermediate pathologists (diagnosis time 3–10 years) D, E, F, senior pathologists (diagnosis time > 10 years) G, H, I) Interpret Ki-67LI through VA, MC, SRC, AI.

**Fig. 4:**
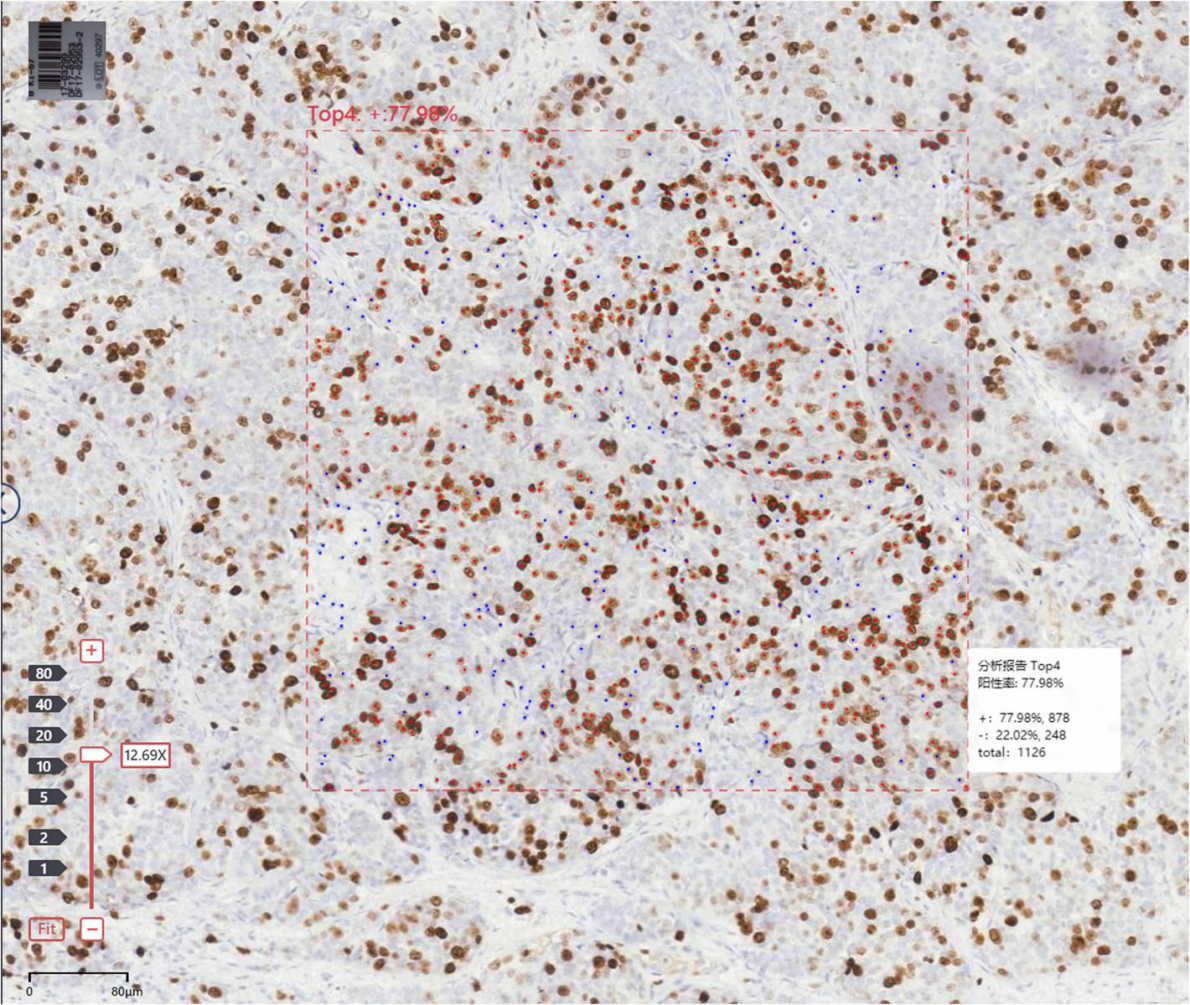
The automatic analysis function of AI software. It can identify positively stained nuclei and negatively stained nuclei, so as to automatically evaluate the expression level of ki67

1. **SRC:** Before interpretation, a pathologist browsed the Ki-67 standard comparison card to form short-term memory. The pathologist browsed the entire slice under a low-power microscope, selected three areas according to the experimental requirements, and then switched to the high-power microscope to perform a rough visual assessment of breast cancer Ki-67 in the three areas at 10% intervals. The Ki-67LI average of the three areas below 30% was evaluated at 5% intervals.
2. **AI:** The AI model is a deep learning network based on Inception V3 and Resnet network.AI analysis software has automatic analysis and frame selection analysis functions, which can identify positively stained nuclei and negatively stained nuclei, so as to automatically evaluate the expression level of ki67(Fig. 4). The pathologist placed three interpretation frames on the digital slice according to the experimental requirements and used artificial intelligence to automatically count and calculate the average value of Ki-67LI in the three interpretation frames.
3. **VA:** The pathologist browsed the entire slice under a low-power microscope, selected three areas according to the experimental requirements, and then switched to the high-power microscope to perform a rough visual assessment of breast cancer Ki-67 in the three areas at 10% intervals. The Ki-67LI average of the three areas below 30% was evaluated at 5% intervals.
4. **MC:** The pathologist browsed the entire slice under the low-power microscope, selected three areas according to the experimental requirements, and manually counted the three areas under a high-power microscope. Finally they calculated the average value.

#### Validation set

A stratified random sampling method was adopted, and one pathologist was randomly selected from the primary, intermediate, and senior pathologists, defined as pathologists X, Y, and Z. They used SRC and AI models to interpret the 150 breast cancer Ki-67 immunohistochemical sections in the validation set. The interpretation method is the same as the SRC and AI interpretation methods in the training set.

### Pathologists and interpretation scenarios

With the same microscope at the same time and place, the pathologist used the same interpretation method for each instance, and the next interpretation was performed after a 2-week forgetting period. The training set was interpreted using VA, MC, SRC, and AI by nine pathologists (junior A, B, C, intermediate D, E, F, senior G, H, I). The verification set adopted stratified random sampling, one (X, Y, Z) was randomly selected from junior, middle, and senior pathologists, and interpreted using SRC and AI. Before starting the interpretation, all pathologists were trained in region selection and AI software.

### Statistical analysis

Interpretation results were analyzed using SPSS 23.0 statistical software. Median and quartile spacing (M ± Q) were used to compare the degree of dispersion of the interpretation method. Kolmogorov-Smirnov (KS) was used for the normality test, intra-group correlation coefficient (ICC) and Bland-Altman scatterplot to check the consistency of interpretation methods. The ICC does not have a unified evaluation standard. According to the definition of the Kappa coefficient [14], the ICC evaluation standard in this experiment was as follows: when it was lower than 0.4, the repeatability was poor; when it was 0.4–0.69, the repeatability was normal; when it was 0.7–0.79, the repeatability was better, when it was above 0.8, the repeatability was very good.

## Results

### Tumor heterogeneity

**Fig. 5:**
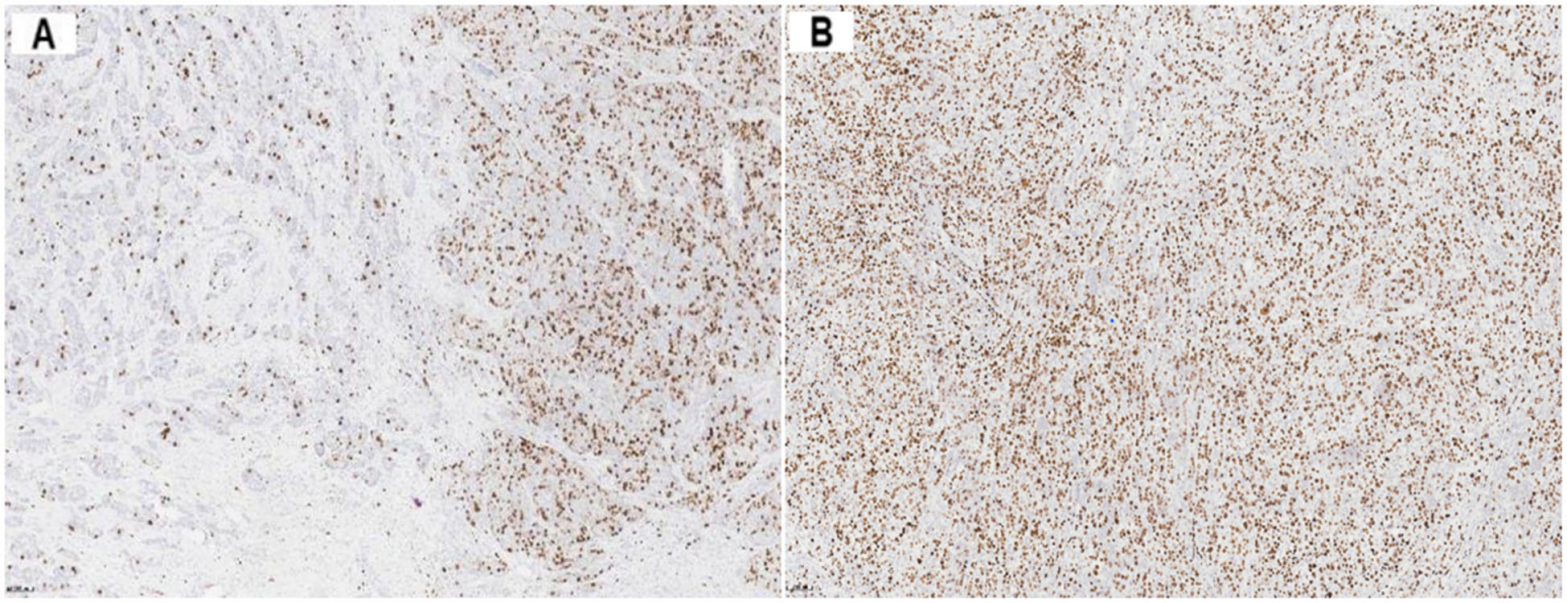
Distribution of breast cancer Ki-67 positive tumor cells (10×). A: Breast cancer Ki-67 positive tumor cells are unevenly distributed, which is heterogeneous. The percentage of Ki-67 positive tumor cells on the left is lower than that on the right (hot spot); B: Ki-67 positive tumor cells of breast cancer are uniformly distributed, showing homogeneity

Due to the heterogeneity of breast cancer, Ki-67 immunohistochemical staining will show uneven distribution of positive tumor cells, that is, hot spots (the area where Ki-67 positive tumor cells are most concentrated). Among the 300 breast cancer immunohistochemical sections in this experiment, 121 (40.33%) cases had no hot spot (homogeneous), and 179 (59.67%) cases had hot spot (heterogeneous). The Ki-67 immunohistochemical sections of breast cancer with and without hot spots are shown in Fig. 5.

### Ki-67LI interpretation time

The results show that the SRC method requires the least time to interpret Ki67 and can improve the work efficiency of pathologists (Table 1). Since the retention time of short-term memory is only 5–20s without retelling, the longest does not exceed 1 min. After the pathologist quickly scans the entire slice and determines the judgment area, he observes and looks for the Ki-67 standard comparison card that is close to the area under the microscope. From this, the Ki-67LI for each area is calculated and the average of the three areas is calculated. The evaluation time for a slice is about 8–30s.

**Table 1:** Time required for interpretation of each Ki-67LI

Interpretation method	VA	MC	SRC	AI
Time(s)	10–40	240–480	8–30	100–120

### Ki-67LI interpretation results

The Kolmogorov-Smirnov (KS) test was used to assess the normality of Ki-67LI in breast cancer using VA, MC, SRC, and AI. Results showed that Ki-67LI in each group did not follow a normal distribution (*p* < 0.05).

**Fig. 6:**
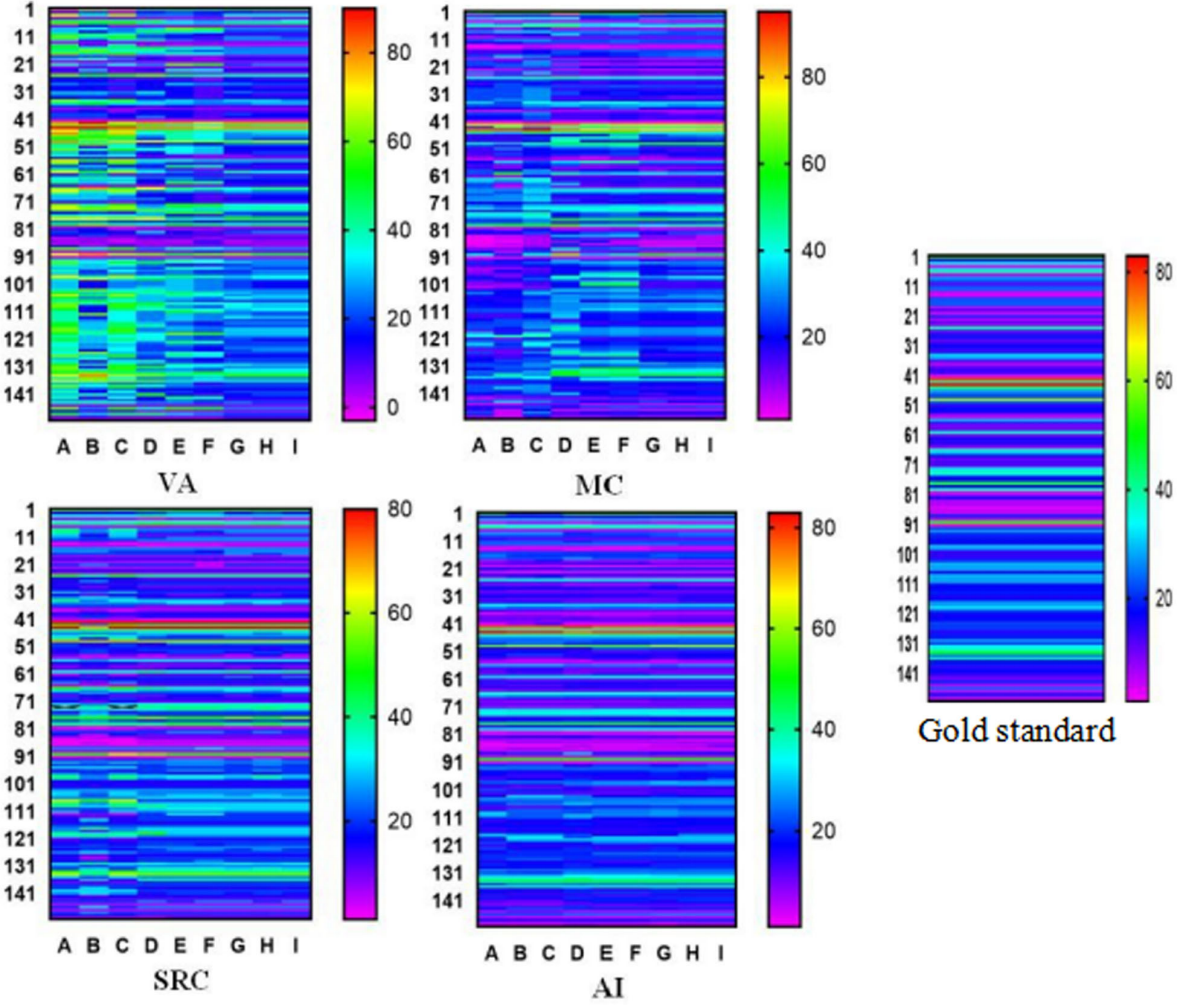
Nine pathologists interpreted breast cancer Ki-67LI heat map through VA, MC, SRC, AI

As shown in Fig. 6, the heat map of nine pathologists interpreting 150 cases of ki67 intuitively shows that the consistency of ki67 interpretation through SRC and AI is higher, and the similarity to the gold standard is also high.

In the training set, using VA, the three junior pathologists (A, B, and C) had poor consistency with the gold standard, and their ICCs were all below 0.80. Among the three intermediate pathologists (D, E, F), the ICCs of the two physicians were all below 0.80. Through MC, intermediate and senior pathologists have good consistency with the gold standard, with an ICC above 0.80, and junior pathologists have poor consistency with the gold standard. Using SRC and AI, the consistency of nine pathologists with the gold standard was very good, with all ICCs above 0.80. In the validation set, using SRC and AI, the three pathologists were in good agreement with the gold standard, with an ICC above 0.95 (Table 2).

**Table 2:** Consistency between the results of nine pathologists’ interpretation of Ki-67LI using VA, MC, SRC and AI and the gold standard

Pathologist	ICC(95%CI)				***P***
	VA	MC	SRC	AI	
A	0.735 (0.652,0.801)	0.764 (0.688,0.823)	0.884 (0.843,0.914)	0.975 (0.965,0.982)	<0.001
B	0.746 (0.665,0.809)	0.753 (0.674,0.815)	0.888 (0.848,0.917)	0.961 (0.946,0.971)	<0.001
C	0.751 (0.672,0.813)	0.764 (0.688,0.823)	0.885 (0.844,0.915)	0.956 (0.940,0.968)	<0.001
D	0.829 (0.772,0.874)	0.883 (0.842,0.914)	0.977 (0.969,0.984)	0.976 (0.966,0.982)	<0.001
E	0.746 (0.688,0.823)	0.860 (0.811,0.896)	0.981 (0.973,0.986)	0.985 (0.979,0.989)	<0.001
F	0.764 (0.689,0.824)	0.878 (0.836,0.910)	0.971 (0.961,0.979)	0.985 (0.979,0.989)	<0.001
G	0.975 (0.965,0.982)	0.983 (0.977,0.988)	0.987 (0.982,0.990)	0.988 (0.983,0.991)	<0.001
H	0.981 (0.974,0.986)	0.982 (0.976,0.987)	0.983 (0.977,0.988)	0.990 (0.986,0.993)	<0.001
I	0.980 (0.973,0.986)	0.982 (0.975,0.987)	0.987 (0.982,0.990)	0.988 (0.983,0.991)	<0.001
X			0.989 (0.985,0.992)	0.994 (0.992,0.996)	<0.001
Y			0.992 (0.990,0.995)	0.995 (0.993,0.996)	<0.001
Z			0.994 (0.992,0.996)	0.995 (0.993,0.996)	<0.001

*P* < 0.05 is statistically significant

*VA* microscopic visual assessment, *MC* microscopic manual counting, *SRC* standard comparison card, *AI* artificial intelligence, *LI* Label Index

In the training set, the consistency of nine pathologists using SRC was significantly higher than that of VA, and this difference was more significant between primary and intermediate pathologists. Consistency comparison with the gold standard shows that the ICC of doctor A after passing SRC increased from 0.735 (0.652, 0.801) to 0.884 (0.843, 0.914); doctor B’s ICC increased from 0.746 (0.665, 0.809) to 0.888 (0.848, 0.917); and doctor C’s ICC increased from 0.751 (0.672, 0.813) to 0.885 (0.844, 0.915) (Table 2).

In the training set, using SRC, the ICC was 0.918 (0.899, 0.936), significantly higher than using VA (ICC was 0.757 [0.711, 0.802]). using AI, the ICC was 0.972 (0.964, 0.978),which is significantly higher than using MC (ICC was 0.803 [0.763, 0.841]). In the verification set, the consistency of the three pathologists was very good using SRC and AI, with ICCs of 0.988 (0.985, 0.911) and 0.990 (0.987, 0.992) (Table 3).

**Table 3:** Consistency analysis of pathologists using VA, MC, SRC and AI to interpret breast cancer Ki-67LI

Variables	ICC(95%CI)		***P***
	Training group	Validation group	
VA	0.757 (0.711,0.802)		<0.001
MC	0.803 (0.763,0.841)		<0.001
SRC	0.918 (0.899,0.936)	0.987 (0.983,0.990)	<0.001
AI	0.972 (0.964,0.978)	0.988 (0.985,0.991)	<0.001

*P < 0.05 is statistically significant

*VA* microscopic visual assessment, *MC* microscopic manual counting, *SRC* standard comparison card, *AI* artificial intelligence, *LI* Label Index

In the homogeneous group of the training set, the inter-observer consistency of VA, MC, SRC, and AI was very good, with ICCs above 0.8. In the heterogeneous group, the inter-observer agreement of the four methods decreased, and the ICC of SRC and AI remained above 0.80. In the homogeneous group of the validation set, the consistency between the pathologists of SRC and AI was very good, with ICCs of 0.985 (0.978, 0.990) and 0.986 (0.979, 0.990). In the heterogeneous group, the agreement between the two pathologists was also very good, with ICCs of 0.990 (0.986, 0.993) and 0.991 (0.986, 0.994) (Table 4).

**Table 4:** The consistency between the training set and the validation set pathologists interpreting breast cancer Ki-67LI in the homogeneous and heterogeneous groups through SRC and AI

Variables	ICC(95%CI)				***P***
	Training group		Validation group		
	Homogeneity	Heterogeneity	Heterogeneity	Heterogeneity	
VA	0.848 (0.785,0.901)	0.698 (0.634,0.760)			<0.001
MC	0.941 (0.914,0.963)	0.672 (0.605,0.738)			<0.001
SRC	0.964 (0.946,0.977)	0.877 (0.844,0.907)	0.985 (0.978,0.990)	0.990 (0.986,0.993)	<0.001
AI	0.984 (0.976,0.990)	0.959 (0.947,0.970)	0.986 (0.979,0.990)	0.991 (0.986,0.994)	<0.001

*P* < 0.05 is statistically significant

*VA* microscopic visual assessment, *MC* microscopic manual counting, *SRC* standard comparison card, *AI* artificial intelligence, *LI* Label Index

## Discussion

Although the results of the study show that a high Ki-67 index is associated with high risk of recurrence and poor survival in patients with early breast cancer [15, 16] and responds well to neoadjuvant chemotherapy [17],because poor reproducibility between observers and the lack of a standardized scoring system [10], its clinical application is still under debate. At present, the factors affecting the poor repeatability of Ki-67 interpretation of breast cancer are mainly the interpretation method and the choice of interpretation area. Ki-67 manual counting method is time-consuming. For each breast cancer Ki-67 immunohistochemical section,the time required for manual counting using a microscope is 10–20 times that required for visual accessments and prone to be affected by visual fatigue, resulting in controversial results. The nine pathologists in the training set of this study showed that the manual counting method under a microscope is more consistent than the cope visual evaluation method, but the manual counting method takes more time and effort. Therefore, manual counting under microscope is not suitable for clinical work. In 2015, Shui Ruohong et al. [7] showed that visual assessment of Ki-67LI at 10% intervals is potentially a standard method in clinical practice of breast cancer treatment. Visual assessment is subjective and susceptible to some factors such as experience. In this study, the Ki-67 standard comparison card was browsed to form an image memory, and then the breast cancer Ki-67LI was evaluated under microscope to discuss the consistency of the visual evaluation before and after the reference comparison card. The results showed that visual assessment (VC) and SRC take less time (Table 1). Regardless of the training set or the verification set, after referring to the Ki-67 standard comparison card, the consistency between pathologists of different levels significantly increased, especially for junior and middle pathologists with insufficient work experience, and less time was required. The image memory method was proposed by the Japanese professor Nanada Shin [18]. The formation of short-term memory through images is currently the most suitable memory method for operation of the human brain. Thus, the Ki-67 standard comparison card can be used as a convenient tool in clinical pathological work.

Digital pathology is an emerging method [19, 20]. Many studies have proposed automatic artificial intelligence analysis as a potentially effective method for Ki-67 evaluation. The results of this study indicate a high degree of consistency for Ki-67LI between artificial intelligence counting and the gold standard. Artificial intelligence for cell counting not only meets the standards of the International Breast Cancer Working Group, but also exceeds the range of cell numbers recommended by the working group. Stålhammar et al. showed that all automatic Ki-67 evaluation methods are far superior to manual evaluation in terms of sensitivity and specificity. However, artificial intelligence software also has some disadvantages. Studies have shown that the automatic evaluation method is less accurate than the visual method in identifying tumor cells. Especially in lymphocyte-rich tumors, some Ki-67 positive lymphocytes may be identified as tumor cells. This causes the Ki-67LI to be overestimated [21]. This study showed that Ki-67LI using AI was lower than by the other three interpretation methods. Owing to the heterogeneity of breast cancer cells, AI cannot completely identify each tumor cell or can misidentify, such as identifying interstitial cells as tumor cells, or neglecting positive tumor cells with blurred outlines and lighter staining, which causes Ki-67LI to be low. This is consistent with the results of the Maeda study, which reported that the average Ki-67LI for visual assessment was 22, and the average Ki-67LI for AI count was 20.4 [22]. In order to overcome this problem, a study has proposed a semi-automatic evaluation method of Ki-67LI, by manually labeling immunostained tumor cells and negative tumor cells to determine the accurate proliferation index value, and then automatically counting the cells. The ratio between the total number of immunolabeled positive cells and the total number of tumor cells is Ki-67LI. The gold standard in this study combined with breast pathology experts and artificial intelligence counting, attempting to avoid computer errors in identifying tumor cells and human interpretation errors due to visual fatigue to reduce the risk of Ki-67LI being overestimated or underestimated.

Because of the heterogeneity of breast cancer tumor cells, most of the Ki-67 immunohistochemical sections of breast cancer have hot spots (high value-added areas) and cold spots (low value-added areas), and the hot spots include tumor margin and central hotspot. The selection of different interpretation areas will inevitably lead to different Ki-67LI results. The International Breast Cancer Ki-67 Working Group suggested that, if there are hot spots in Ki-67 breast cancer immunohistochemical staining sections, the choice of interpretation area should include hot spots [3]. The four methods for interpreting Ki-67 in breast cancer in this experiment selected a hot spot area and two non-hot spot areas (including the lowest value-added area of the entire slice and the area in between),which overcomes the influence of the selection of different evaluation areas on the repeatability of Ki-67 immunohistochemical interpretation of breast cancer. The results show that in heterogeneous tumors, artificial intelligence counting can minimize the impact of tumor heterogeneity on Ki-67LI, and significantly increase the consistency among pathologists. However, in the difference test with the gold standard, there was a difference between the average value of Ki-67LI for breast cancer interpretation using SRC, AI, and the gold standard. This difference may be caused by the choice of interpretation area. In the whole Ki-67 immunohistochemical section, three regions were selected in the experimental method; therefore, multi-region analysis can still be performed to test the difference among each one of them and the gold standard. From this point of view, artificial intelligence software for breast cancer Ki-67LI tends to standardize the interpretation area (multi-area average method), which is very important for the clinical application of Ki-67.

The International Breast Cancer Ki-67 Working Group (IKWG) published an update of Ki-67 assessment, discussing the analytical validity and clinical application status of Ki-67 immunohistochemical detection in breast cancer tissues, and recommends the use of standardized visual assessments method [23]. However, due to time and human factors, we will do breast cancer ki67-related research based on the new consensus in another article later.

In short, it is an important task for pathologists to determine the standardized method of Ki-67 interpretation of breast cancer. Artificial intelligence software has high accuracy and repeatability in the interpretation of breast cancer Ki-67 immunohistochemistry. In some pathology laboratories, where artificial intelligence software has not yet been popularized, breast cancer Ki-67 is interpreted with reference to the breast cancer Ki-67 standard comparison card to ensure repeatability of the interpretation results with the premise of saving time and effort. Therefore, Ki-67 standard comparison card is expected to become a reference method for the daily interpretation of Ki-67 immunohistochemical results of breast cancer.

## Conclusions

AI has satisfactory inter-observer repeatability, and its true value is closer to that of the gold standard, which is the preferred reproducibility method of Ki-67LI.While AI software is not yet popular, SRC may be a standard candidate interpretation method for breast cancer Ki-67LI.

## Data Availability

All data generated or analysed during this study are included in this published article.

## Abbreviations

Ki-67LI: Ki-67 Label Index
ICC: Intraclass correlation coefficient
95%CI: 95% Confidence Interval
ER: Estrogen Receptor
PR: Progesterone Receptor
VA: Visual Assessment
MC: Manual Counting
SRC: Ki-67 Standard Reference Card
AI: Artificial Intelligence Counts
HER-2: Human Epidermal Growth Factor-2
M Ki-67: Marked Ki-67
